# Analysis of a vascular screening program in a rural community

**DOI:** 10.4103/0975-3583.64444

**Published:** 2010

**Authors:** Luiz F. Galvao, Monica Pierri-Galvao

**Affiliations:** *Scranton Primary Health Care, 959 Wyoming Ave, USA*; 1 *Marywood University, Department of Science, 2300 Adams Ave, Scranton, PA 18509, USA*

**Keywords:** Atherosclerosis, screening, vascular

## Abstract

**Background:**

Implementation and adaptation of a vascular screening program in a rural community.

**Methods and Results:**

A vascular screening program was offered free to the general population aged 55 years and older. It comprised of ultrasound screening of the carotid arteries, abdominal aorta, and the lower extremity segmental Doppler with ankle brachial index measurements. The program was initially developed in Annapolis, MD, and adapted to rural Warren, PA. Between March 2008 and June 2009, a total of 758 screenings were completed. Mild disease was detected in 12.7% of the population, moderate disease in 2.5% of the population, and severe disease in 1.3% of the population; 61.7% of all the participants had atherosclerotic plaques without stenosis and 45.9% of the participants had a history of smoking, 12.1% diabetes, 81.4% dyslipidemia, 58.3% hypertension, and 9.8% heart disease.

**Conclusion:**

This model of vascular screening program is an important tool for the detection of vascular disease and preventive health counseling. It detects not only vascular disease but its associated risk factors. Adequate treatment decreases cardiovascular disease mortality. This program, through local sponsorship, is adaptable to the rural community.

## INTRODUCTION

Data from the National Vital Statistics reports show that 26% of the total deaths in the USA are due to heart disease and 5.7% are due to cerebrovascular disease.[1] Death due to heart disease is caused mainly by atherosclerotic obstruction of the coronary arteries. Atherosclerosis is a systemic disease affecting not only the coronary and carotid arteries but also the abdominal aorta and the lower extremities. The correlation of death and atherosclerosis of the carotid arteries and lower extremity arteries is not straightforward as in the coronaries. Therefore, screening for carotid disease and peripheral artery disease (PAD) in an asymptomatic population is controversial. Screening for abdominal aorta aneurysm (AAA) is well established and reduces mortality from ruptured aneurysm.

Screening for vascular disease would make sense if we could impact on decreasing deaths and morbidity from coronary artery disease (CAD), stroke, aortic aneurysm rupture, and limb loss. It also needs to be cost effective.[2] The current screening recommendation for vascular disease is restricted to AAA in men older than 65 years with a history of smoking.[3‐5] The data show that screening for asymptomatic carotid disease and PAD does not impact on decreasing the incidence of stroke death or of loss of limb, but it does correlate with finding a population with high cardiac mortality in which intervention would have an impact.[6‐9]

In our study population, we provided ultrasound of the carotid arteries, abdominal aorta, and segmental Doppler of the lower extremities. After screening 758 people aged 40 years or older, we found 61.7% with atherosclerotic plaques without stenosis, 12.7% with mild disease, 2.5% with intermediate disease, and 1.3% with severe disease. The definition of mild, intermediate, and severe disease is given in Table 1. This service was modeled after the Dare to CARE program in Annapolis, MD.[10]

**Table 1:** Disease severity

	Carotid obstruction (%)	Abdominal aortic aneurysm (cm)	PAD (ABI)
Mild	1–39	3–3.9	0.7–0.95
Intermediate	40–59	4–4.9	0.5–0.69
Severe	≥60	≥5	≥0.5

*ABI, ankle brachial index; PAD, peripheral artery disease*.

## MATERIALS AND METHODS

We offered free vascular screening for the general population aged 55 years and older. Younger people were not rejected (which explains the participation of younger individuals). We had a group of 1 ultrasound technician, 1 secretary coordinator, 1 nurse practitioner, and 3 physicians. The results were discussed with each participant and a report was sent to their primary care physician . Any severe or critical result was referred to our vascular surgeon who would see the patient promptly.

The program is offered in a 2-day session: On day 1, there is a lecture on vascular disease, including prevention, diagnosis, and treatment. Participants receive a voucher for a free laboratory test, including lipid profile and fasting glucose. The lecture is limited to 200 participants. On day 2, there are blood pressure measurement with ankle brachial index (ABI) calculation, and the ultrasound screening with the results annotated by the technician. The ultrasound is done 3 times a week until all 200 participants have had their tests done. The nurse practitioner discusses all the data with the participant and advises on prevention and treatment, including lifestyle change, smoking cessation, obesity, use of antiinflammatory drugs (eg, aspirin), and statins or possible surgical intervention.

The diagnostic criteria and interpretation of carotid stenosis follows the Dare to CARE program and is based on the Commission for the Accreditation of Vascular Laboratories [Table 2].[11]

The screening is now offered every 3 months. It is totally free and sponsored by a local foundation and the local community hospital. It took the team about 4 months to become proficient. The hospital offers 2 rooms for the day 2 session. The lecture is presented elsewhere at a local auditorium.

All the physicians involved in the lectures (2 internal medicine physicians and 1 vascular surgeon) volunteer their time. The vascular technician, the nurse practitioner, and the secretary coordinator work during their regular shifts. They are employed by the hospital. The secretary coordinator also organizes the evening lectures.

**Table 2:** Carotid duplex velocity interpretation criteria

Category	PSV lower	Upper	EDV lower	Upper	ICA/CCA lower	Upper
Normal	0.1	109.9	0	39.9	0	1.4
Mild: 1%–39%	110	129.9	0	39.9	1.5	2.9
Moderate: 40%–59%	130	169.9	0	39.9	1.5	2.9
Severe: 60%–79%	170	249.9	40	99.9	3	5.9
Critical: 80%–99%	250	999.9	100	999.9	6	99.9
Occluded	0	0	0	0	0	0

*PSV, peak systolic velocity; EDV, end diastolic velocity; ICA/CCA, internal carotid artery/common carotid artery velocity ratio*,

## RESULTS

A total of 758 people were screened. Of these, we have data on age, gender, lipid profile, fasting blood glucose, blood pressure, ultrasound, and ABI of 627 participants. The ultrasound results and ABI data were available for all the 758 participants. Some data (age, gender, lipid profile, fasting blood glucose, and blood pressure) were missing from the first 131 participants when we were learning and adapting the program.

All participants live in Warren County, PA. They were in the age group of 40–99 years and 62% were women.

There were 45.9% with history of smoking, 12.1% with diabetes, 81.4% with dyslipidemia, 58.3% with hypertension, and 9.8% with heart disease.

Mild disease (carotid stenosis ≤ 39%, 0.7 ≤ ABI ≤ 0.95 or 3.0 cm ≤ AAA ≤ 3.9 cm) was detected in 12.7% of the population; moderate disease (carotid stenosis 40–59%, 0.5 ≤ ABI ≤ 0.69 or 4.0 cm ≤ AAA ≤ 4.9 cm) in 2.5% of the population and severe disease (carotid stenosis ≥ 60%, ABI < 0.5 or AAA ≥ 5.0 cm) in 1.3% of the population; 2.6% of the population with vascular disease had no risk factors, such as smoking, diabetes, dyslipidemia, and hypertension. As expected, the presence of these risk factors increased the prevalence of moderate and severe vascular disease by 56%, 32%, 444%, and 139%, respectively; 61.7% of all the participants had atherosclerotic plaques without stenosis. The results of carotid disease, AAA and PAD are summarized in Tables 3, 4, and 5. Table 6 shows the detection of any vascular disease as a group.

**Table 3:** Testing results—carotid disease detection

Age group (N)	Mild: 1–39%		Moderate: 40–59%		Severe: ≥ 60%	
	%	n	%	n	%	n
40–49 (35)	8.6	3	0.0	0	0.0	0
50–59 (187)	6.4	12	1.6	3	0.5	1
60–69 (225)	8.0	18	2.2	5	0.9	2
70–79 (127)	10.2	13	6.3	8	2.4	3
80–89 (51)	5.9	3	3.9	2	0.0	0
90–99 (2)	0.0	0	0.0	0	0.0	0
All (627)	7.8	49	2.9	18	1.0	6
Including extras (n = 131)						
758	7.1	54	2.5	19	1.1	8

**Table 4:** Testing results—AAA

Age group (N)	3 cm ≤ AAA ≤ 3.9 cm		4 cm ≤ AAA ≤ 4.9 cm		AAA ≥ 5 cm	
	%	n	%	n	%	n
40–49 (35)	0.0	0	0.0	0	0.0	0
50–59 (187)	1.1	2	0.0	0	0.0	0
60–69 (225)	0.4	1	0.0	0	0.0	0
70–79 (127)	1.6	2	1.6	2	0.0	0
80–89 (51)	2.0	1	0.0	0	0.0	0
90–99 (2)	0.0	0	0.0	0	0.0	0
All (627)	1.3	6	0.3	2	0.0	0
Including extras (N = 131)						
758	1.1	8	0.3	2	0	0

*AAA, abdominal aorta aneurysm*.

**Table 5:** Testing results—PAD

Age group (N)	0.7 ≤ ABI ≤ 0.95		0.5 ≤ ABI ≤ 0.69		ABI < 0.5	
	%	n	%	n	%	n
40–49 (35)	2.9	1	0.0	0	0.0	0
50–59 (187)	8.0	15	0.5	1	0.0	0
60–69 (225)	7.6	17	0.0	0	0.0	0
70–79 (127)	10.2	13	2.4	3	0.8	1
80–89 (52)	5.9	3	2.0	1	2.0	1
90–99 (2)	0.0	0	0.0	0	0.0	0
All (627)	7.8	49	0.8	5	0.3	2
Including extras (N = 131)						
758	7.3	55	0.9	7	0.3	2

*ABI, ankle brachial index, PAD, peripheral artery disease*.

**Table 6:** Vascular disease detection

All Age groups (N)	Plaque only		Mild disease		Intermediate disease		Severe disease	
	%	n	%	n	%	n	%	n
627	58.4	366	13.9	87	2.9	18	1.3	8
Including extras (N = 131)						
758	61.7	468	12.7	96	2.5	19	1.3	10

## DISCUSSION

The detection of atherosclerotic plaques in 61.7% of the population was found to be significant. These data alone can be used to address lifestyle change and risk factor modification. Plaque, carotid disease, and carotid artery intima thickness are known to increase cardiovascular mortality.[7,8,12,13] PAD is also strongly correlated with CAD[14] As a preventive measure, this program will probably have an impact on the incidence of vascular diseases and mortality. We hope that advocating the use of statins will reverse the progression of atherosclerosis in carotid arteries.[15] The education provided by this program will also decrease the incidence of smoking and obesity. On the long run it may also improve diseases, such as diabetes, hypertension, and dyslipidemia. A few patients will benefit immediately from the detection of severe cases in which surgery may be indicated to prevent stroke, AAA rupture, or limb loss.

Carotid stenosis is responsible for only about 20% of ischemic strokes; therefore, more sophisticated tests, such as transcranial Doppler for the identification of microemboli may identify high-risk individuals for more aggressive intervention.[9,16]

Our data showed that compared with Annapolis, MD (population 36,000), the rural town of Warren, PA (population 10,000), had a similar incidence of vascular disease. In Annapolis the incidence of severe carotid disease was 1.7% vs. 1.1% in Warren, PAD 6.9% vs. 8.4% and AAA 2.2% vs. 1.3%, respectively. Therefore, the impact of such interventions does not appear to change from urban to rural communities. The Dare to CARE program could be implemented in other cities and be sponsored by local foundations, hospitals, and health insurance groups. Although we did not calculate the cost of such a program, it has been shown elsewhere to be $25.00 per participant.[10]

The Dare to CARE vascular screening program is a useful tool in preventive medicine. Ultrasound not only detects vascular disease but it gives people raw data on which they can work to prevent and control diseases, such as diabetes, hypertension, dyslipidemia, and atherosclerosis. Although screening asymptomatic people for carotid disease and PAD is controversial, it does show a high-risk population for cardiac death in which intervention is advisable. The program is adaptable to the rural setting.
